# Distal Upper Limb Injuries in Skiing and Snowboarding: A Two-Season Study from a High-Volume Trauma Center in the Italian Dolomites

**DOI:** 10.3390/medicina61101787

**Published:** 2025-10-03

**Authors:** Michele Paolo Festini Capello, Nicola Bizzotto, Fjorela Qordja, Svea Misselwitz, Chiara Sernia, Salvatore Gioitta Iachino, Giuseppe Petralia, Valerie A. A. van Es, Pier Francesco Indelli, Christian Schaller

**Affiliations:** 1 Department Orthopaedic Surgery, Südtiroler Sanitätsbetrieb (SABES), Dantestraße 51, 39042 Brixen, Italysalvatore.gioittaiachino@sabes.it (S.G.I.); pindelli@stanford.edu (P.F.I.);; 2 Clinic of Orthopaedics, Department of Clinical and Molecular Science, Università Politecnica delle Marche, Via Tronto 10/a, 60126 Ancona, Italy; qordja.fjorela@gmail.com; 3 Institute of Biomechanics, Paracelsus Medical University (PMU), 5020 Salzburg, Austria; 4 Minivasive Orthopaedic Surgery, Department of Life, Health & Environmental Sciences, University of L’Aquila, 67100 L’Aquila, Italy; 5 MoMiLab Research Unit, IMT School for Advanced Studies Lucca, 55100 Lucca, Italy; valerie.vanes@imtlucca.it

**Keywords:** skiing, snowboarding, wrist fractures, skier’s thumb, upper extremity injuries, injury prevention

## Abstract

*Background and Objectives:* Distal upper limb injuries are frequent in winter sports, but their functional impact is often underestimated. This study aimed to describe the epidemiology, mechanisms, and risk factors for injuries involving the forearm, wrist, hand, and fingers sustained during two consecutive winter seasons in the Italian Dolomites. *Materials and Methods:* All adult and willing patients presenting to the Emergency Department of Brixen Hospital after ski- or snowboard-related accidents between December 2023 and March 2025 completed a standardized 23-item questionnaire on demographics, experience level, environmental factors, equipment, and trauma mechanism. For the aim of this study only distal upper limb injuries were extracted and analyzed. Statistical analyses compared fracture versus non-fracture injuries, “good” versus “bad” fractures (AO classification and surgical complexity), and isolated ulnar collateral ligament (UCL) injuries. *Results:* A total of 195 patients were analyzed: 96 (49.2%) sustained a fracture and 33 (16.9%) presented with isolated UCL lesions. Fractures occurred more frequently on blue slopes (56.2% vs. 33.3%, *p* < 0.001), whereas non-fracture injuries predominated on red and off-piste slopes. Age, BMI, and skill level did not differ significantly between groups. Surgically classified complex distal forearm fractures were significantly more frequent in females (*p* < 0.005) but were not associated with environmental factors. UCL injuries occurred mainly on red slopes (54.5%) and were often related to pole entrapment during falls. None of the injured patients reported the use of protective wrist or thumb supports. *Conclusions:* Distal upper limb injuries are a common pattern of alpine sports trauma, with wrist fractures and skier’s thumb being predominant lesions. Low-speed falls on easy slopes are associated with wrist fractures, while UCL injuries are linked to intermediate slopes. Preventive strategies should include fall technique education, protective gloves, and improved pole ergonomics.

## 1. Introduction

Skiing and snowboarding are highly popular sports in the Italian alpine region of South Tyrol. According to the Provincial Institute of Statistics of Bozen (ASTAT), about 11,000 ski- and snowboard-related accidents occurred during the 2022–2023 winter season in the province of Bozen, Italy, a figure that remains consistent with previous years despite post-pandemic fluctuations in tourism and slope attendance [1,2,3,4,5].

Ski- and snowboard-related injuries have been extensively studied, especially those affecting the lower limbs. The knee is the most common affected region and in particular the anterior cruciate ligament (ACL) and medial collateral ligament (MCL) sprains are among the most common injuries, representing 10–33% of all cases in the literature [6,7,8,9,10,11,12]. However, injuries to the upper extremities, particularly isolated trauma from the forearm to the hand, are still frequently observed and retain considerable epidemiological importance despite technological advancements [13] with studies reporting incidences of up to 19% of all ski related and up to 47% of all snowboard related injuries [14].

These injuries are especially relevant in skiing and snowboarding falls, where reflexive arm extension, incorrect pole use, or impact absorption mechanisms often cause trauma to the distal upper extremity [15,16]. Additionally, upper limb injuries may follow different epidemiological patterns compared to lower limb injuries, potentially influenced by factors such as age, skill level, and the specific mechanics of the fall or collision [17,18].

A more detailed understanding of this injury patterns could help inform future prevention strategies and resource allocation in high-volume trauma centers in alpine regions.

The objective of this study was to analyze all ski- and snowboard-related injuries involving the distal upper extremity (from the forearm to the fingers) collected at a single high-volume trauma center during the 2023/2024 and 2024/2025 winter seasons. The authors aimed to identify common injury patterns, associated demographic and environmental factors, and potential risk profiles specific to these types of lesions. Particular attention was paid to distal forearm fractures and lesions of the ulnar collateral ligament (UCL) of the thumb’s metacarpophalangeal joint (MCPJ), often referred to as “skier’s thumb”.

## 2. Materials and Methods

### 2.1. Study Design and Setting

**Figure A1:**
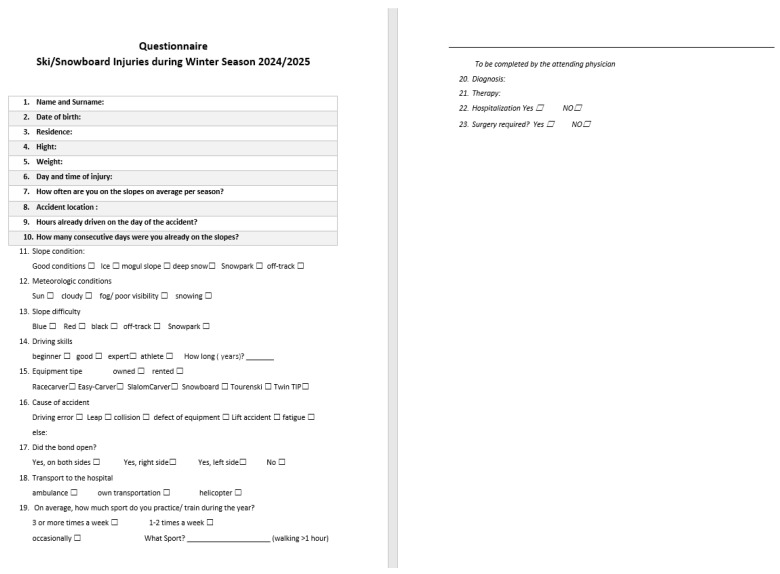
Validated 23-item questionnaire for skiing or snowboarding accidents.

During the winter seasons of 2023/2024 and 2024/2025, all adults presenting to the emergency department at Brixen Hospital in South Tyrol, Italy, after skiing or snowboarding accidents were invited to complete a standardized 23-item questionnaire (Appendix A Figure A1). The questionnaire was specifically developed by the authors and has already been used in a previous study [13]. Data collection occurred from 7 December 2024 to 31 March 2025.

### 2.2. Questionnaire and Data Collection

The survey, available in Italian, German, and English, was designed to gather detailed information in three main areas: personal and clinical background (including age, sex, BMI, comorbidities, medications, and skiing experience), environmental conditions at the time of injury (such as slope type, snow and weather conditions, altitude, time of day, and equipment used), and injury-related characteristics (mechanism of trauma, body part involved, and transport to the hospital).

Questionnaires were distributed in the waiting area or, in cases requiring urgent care, completed after the patient had been stabilized. Emergency and orthopedic physicians made diagnoses based on clinical, radiographic, and ultrasound assessments. Advanced imaging, such as MRI, was reserved for selected cases during outpatient follow-up and was not routinely available in the acute setting.

For this prospective analysis, all adult patients (≥18 years) who sustained injuries to the distal upper limb (forearm, wrist, hand, or fingers) while skiing and who agreed to fill out the questionnaire were included. Eligible patients had a confirmed diagnosis based on clinical examination and standard radiographs. Exclusion criteria were: age under 18 years, injuries not related to alpine skiing, inability to understand or complete the questionnaire accurately, and incomplete or missing data on key study variables.

### 2.3. Variable Definitions and Categorization

Data were categorized according to

- Injury type (e.g., fractures, ligamentous injuries)
- Injury location (e.g., forearm, wrist, hand, fingers)
- Environmental context (e.g., slope type, snow conditions, weather)
- Equipment characteristics, and
- Skier demographics

### 2.4. Statistical Analysis

Descriptive statistics summarized demographic, environmental, and injury-related variables. Continuous variables are reported as mean ± standard deviation (SD), while categorical variables are shown as counts and percentages (*n*, %). Analyses were performed in three stages.

#### 2.4.1. Fracture vs. Non-Fracture Risk Analysis

All lower-arm injuries (forearm, wrist, hand, and fingers) were classified as either fractures or non-fractures. Comparisons were made between these groups to identify potential risk factors for sustaining a fracture.

Continuous variables were tested for normality using the Lilliefors test. Depending on the distribution, group differences were evaluated with either independent *t*-tests (for normally distributed variables) or Mann–Whitney U tests (for non-normal distributions). Categorical variables were compared using Pearson’s chi-square or Fisher’s exact tests, as appropriate.

#### 2.4.2. Risk Factors for Fracture Severity (Good vs. Bad)

Within the fracture group, injuries were further classified as “Good” (non-complex, non-surgical) or “Bad” (complex or requiring surgery). “Good” injuries mostly corresponded to AO classification 2R3A, 2R3B1, and 2R3B2, while “Bad” injuries included 2R3B3, all type C fractures, and cases with associated ulnar fractures. Comparisons between these subtypes were performed to identify clinical and environmental predictors of injury severity. Statistical testing followed the same approach as above: continuous variables were tested for normality and compared using either *t*-tests or Mann–Whitney U tests, and categorical variables were evaluated using chi-square or Fisher’s exact tests.

#### 2.4.3. UCL Injury Subgroup Analysis

In the third-level analysis, we isolated cases with UCL thumb lesions to examine their clinical and environmental features. Descriptive statistics summarized demographics, injury context, and equipment use.

Categorical variables were analyzed with the chi-square (χ^2^) goodness-of-fit test to evaluate deviations from a uniform distribution; omnibus *p*-values were reported. For continuous variables (e.g., age, BMI, activity exposure, driving experience), we tested for normality using the Lilliefors test. Group comparisons were conducted using Welch’s *t*-test, and results were reported as mean ± SD. All tests were two-tailed, with *p* < 0.05 considered statistically significant, accommodating unequal variances between male and female participants.

Analyses were performed using MATLAB R2024b.

## 3. Results

A total of 195 patients were included in the upper arm injury database. Among them, 96 sustained a fracture, while 99 did not have a fracture. Of the 96 fractures, 47 were classified as “good” fractures (i.e., surgically straightforward), and 16 were considered “bad” fractures (i.e., difficult for surgical intervention). Additionally, 33 patients had an isolated UCL injury without any evidence of fracture.

The final cohort (Table 1) included 92 males (47.2%) and 103 females (52.8%), with a mean age of 40.4 ± 17.7 years and a mean BMI of 23.5 ± 3.7 kg/m^2^. On average, patients reported 9.1 ± 10.8 skiing or snowboarding days per season and had been active for 2.4 ± 1.7 h on the day of injury. Regarding skill level, 48.7% self-identified as good skiers or snowboarders, 26.7% as experts, 21.5% as novices, and 3.1% as professional athletes.

As shown in Table 2, most injuries occurred on slopes of intermediate (red) difficulty (40.5%), followed by easy (blue) slopes (44.6%) and difficult (black) slopes (9.7%). A minority of incidents occurred off-piste (4.6%) or in snow parks (0.5%). The majority of accidents occurred on well-prepared slopes (66.2%), with fewer reported on icy (14.4%) or bumpy (13.8%) terrain. Weather conditions were favorable in most cases, with 69.2% of accidents occurring on sunny days and 20.0% on cloudy days; only 4.6% occurred during fog or poor visibility and 6.2% during snowfall.

Equipment characteristics are detailed in Table 2. Slightly more than half of the patients (58.5%) were using rented equipment, while 41.5% used their own. The most frequently used equipment types were all-round/easy-carver skis (35.9%), race-carver skis (23.6%), and touring skis (17.4%), followed by slalom-carvers (9.2%), other types (10.3%), and twin-tip skis (2.1%). Only one patient (0.5%) was using a snowboard at the time of injury.

As reported in Table 3, the most common self-reported cause of injury was a mistake (65.6%), followed by collision (14.9%) and fatigue (5.6%). Only 6.7% of injuries occurred during jumps. Binding release occurred in 31.8% of cases. Nearly half of the injuries affected the wrist (49.7%), followed by the hand (35.4%) and fingers (13.3%). Laterality was nearly balanced, with 49.7% of injuries occurring on the left side and 50.3% on the right.

### 3.1. Fracture vs. Non-Fracture Risk Analysis Results

An analysis of baseline patient characteristics showed no significant differences between the fracture (*n* = 96) and non-fracture (*n* = 99) groups in terms of age (38.7 ± 17.1 vs. 42.1 ± 18.2 years, *p* = 0.183), BMI (both groups: 23.5 ± ~3.7 kg/m^2^, *p* = 0.988), or skiing/snowboarding activity levels (Table 4). Sex distribution was balanced, and self-rated driving skills indicated a trend toward higher fracture rates in less experienced individuals (*p* = 0.090).

The environmental context varied significantly between groups. Fractures happened more often on easier slopes (blue: 56.2% vs. 33.3%, *p* < 0.001), while non-fractures were more common on red and off-piste terrains. Snow quality and weather conditions did not differ significantly between groups (Table 5). The type of equipment showed a borderline difference (*p* = 0.0637), with more fractures in users of race carvers and fewer among all-round/easy-carver users.

Accident mechanisms were mostly similar between groups, with individual errors being the main cause (68.8% fractures versus 62.6% non-fractures, *p* = 0.5692). Binding release did not differ significantly (*p* = 0.4377), although the type and site of injury varied greatly (*p* < 0.001 for both) (Table 6).

### 3.2. Risk Factors for Fracture Severity (Good vs. Bad) Results

Among patients with fractures, 47 were classified as “good” (AO classification 2R3A, 2R3B1, and 2R3B2) and 16 as “bad” (AO classification 2R3B3, all type C fractures, and cases with associated ulnar fracture). No significant differences in age (35.8 ± 17.5 vs. 40.1 ± 11.7 years, *p* = 0.269), BMI (23.2 ± 3.4 vs. 23.5 ± 3.1 kg/m^2^, *p* = 0.783), or activity levels were observed between groups (Table 7). However, sex distribution showed a significantly higher proportion of females in the bad fracture group (75.0% vs. 53.2%, *p* < 0.005).

Environmental conditions and equipment variables showed no significant link to fracture severity. Both groups mostly sustained injuries on blue or red slopes in sunny or cloudy weather, using either rented or owned gear (Table 8). Similarly, slope conditions and equipment type did not seem to affect the complexity of fractures.

The cause of injury was similar for both good and bad fractures, mostly attributed to individual errors (78.7% vs. 75.0%, *p* = 0.8518). (Table 9).

### 3.3. UCL Injury Subgroup Analysis Results

Thirty-three patients sustained isolated UCL injuries without any signs of fracture. The average age in this group was 43.2 ± 16.8 years, with a slight female predominance (51.5%). These patients reported slightly higher activity levels compared to the overall group, skiing for 2.9 ± 1.7 h per day over 10.1 ± 8.5 days (Table 10). Most identified as good (60.6%) or expert (30.3%) skiers, with only 9.1% being novices.

UCL injuries were more frequently sustained on red slopes (54.5%) under well-prepared snow and sunny conditions (63.6%) (Table 11). The most common cause was self-inflicted error (72.7%), although jumps accounted for 15.2% of cases—significantly more than in the fracture groups. Equipment use did not differ notably, but most patients used rented all-around skis.

Although binding release occurred in 36.4% of UCL injuries, it was not significantly different from other groups. The injury laterality was relatively balanced, with 57.6% on the right and 42.4% on the left (Table 12).

## 4. Discussion

This two-season analysis provides new insights into the epidemiology and mechanisms of distal upper limb injuries in skiing and snowboarding.

### 4.1. Injury Prevalence

Attention should be paid to upper limb injuries in skiing and snowboarding, as in our cohort, they accounted for a considerable burden, with a total of 195 patients—96 presenting with fractures and 99 with non-fracture injuries. No significant differences in upper limb injuries emerged in terms of sex or practiced activity (skiing vs. snowboarding).

Our findings are consistent with the recent meta-analysis by Chauffard et al. [19], which synthesized data from more than 750,000 cases, confirming that upper extremity injuries account for a substantial proportion of ski and snowboard trauma, with the wrist predominating in snowboarding (35% of all cases) and the hand in skiing (18%), second only to shoulder trauma (37%). Data from the American National Trauma Data Bank are consistent, as it emerges that 19% of the injuries after skiing and snowboarding accidents lead to upper extremity injury, followed by lower limb injuries in 17.1% and by pelvic fractures in 8% [7]. On the contrary, in a comprehensive review performed by Davey et al. [10], bony injuries to the elbow and fractures of the mid-and distal humerus, radius, and ulna were uncommon. By far the most common is injury to the UCL, although it is frequently underreported due to the patients’ perception that this injury is not serious.

As for gender-specific differences in skiing accidents, a retrospective registry analysis within the Austrian National Registry of Mountain Accidents revealed that men were more often involved in accidents caused by falling or obstacle impact while on slopes with higher difficulty levels. Women more often sustained injuries on easier slopes and while standing or sitting [20].

When analyzing sport activity practiced, Telgheder et al. [21] the rate of upper extremity injury is higher in snowboarders than in skiers.

### 4.2. Trauma Mechanism

A key finding of our work was that upper-limb fractures occurred more often on easier slopes, while non-fracture injuries were more common on red and off-piste slopes. This pattern probably reflects different fall dynamics rather than the terrain’s inherent difficulty. On blue slopes, falls tend to happen at lower speeds or even from a standing position; in these cases, skiers often have time to react, extend their arms, and try to break their fall with their hands. This “protective reflex” is well-known as a main cause of distal radius fractures in low-energy falls because the impact force is transmitted axially through the outstretched hand [15,16]. Conversely, falls on steeper or faster slopes are usually more sudden and uncontrolled. In such situations, there is less time for a protective reaction, and the fall tends to be more random and chaotic, with fractures typically occurring in other anatomical sites. On these red slopes, skier’s thumb was particularly frequent among the upper-limb injuries, as valgus forces on the thumb may be generated when the ski pole is caught in the snow or firmly held during a fall [22,23].

This difference in fall biomechanics could explain why wrist fractures mainly occurred on easy slopes in our series, while injuries without fractures or without major involvement of the distal forearm were more frequent at higher speeds.

### 4.3. Risk Factors

As already shown, we can consider falls on blue slopes as a risk factor for upper limb fractures, while skiing on red slopes is more often associated with soft tissue injuries.

Furthermore, our analysis revealed that female sex was a significant risk factor for complex (“bad”) fracture patterns. In fact, we noticed that fracture severity was not linked to environmental or equipment type but to female sex (*p* < 0.005). Similar sex differences have been reported in distal radius fractures, suggesting that sport activity acts mainly as a triggering event, while underlying bone structure or hormonal factors predispose female patients to complex fracture patterns. Indeed, Helmig et al. also reported that fractures around the wrist were most common among women in their cohort, followed by young riders and beginners [24].

Environmental factors did not emerge as a risk factor for upper limb injuries. Indeed, the majority of injuries (69.2%) occurred on sunny days and, similarly, 66.2% of injuries happened on well-prepared slopes (66.2%). Actually, the most common self-reported cause of injury was a mistake (65.6%), followed by collision (14.9%) and fatigue (5.6%). Conversely, other authors have emphasized the role of environmental conditions, particularly snowfall and snowpack, as significant determinants of injury rates, suggesting that prevention strategies should also address these factors [25]. In this regard, hard-packed snow or icy conditions have been shown to aggravate the risk of UCL injuries [10].

The use of protective equipment was not observed in our cohort; in fact, none of the patients reported using protective gear such as wrist guards or reinforced gloves. Although such equipment has been shown in other sports (especially snowboarding) to lower the risk of fractures and ligament injuries of the wrist and thumb, its use remains controversial in modern literature [26]. The use of specialized gloves with thumb stabilizers and quick-release pole straps has been proposed as a potential preventive measure [24]; however, more targeted kinematic and biomechanical studies will be needed to determine their actual effectiveness.

### 4.4. Preventive Strategies

Based on these findings, prevention strategies should target not only high-risk athletes on steep slopes but also novice and intermediate skiers, who are more likely to experience low-speed falls with bracing. Educational programs that emphasize safe fall techniques—such as avoiding bracing with the hands and releasing poles—and encourage the use of protective gear like gloves or wrist guards may help reduce these injuries. Specifically, for UCL injuries, rethinking pole design and strap use—favoring quick-release systems—could lessen the forces transmitted to the thumb during a fall. Lastly, since complex fractures are more common among women, awareness campaigns and tailored preventive advice for female skiers could be especially beneficial.

### 4.5. Study Limitations

This study has several limitations. First, we focused exclusively on patients older than 18 years, in accordance with the approval of the regional ethics committee; therefore, we are not able to define the epidemiology and injury patterns in children and adolescents. Second, the lack of routine MRI may have led to an underestimation of ligamentous injuries; however, in the emergency setting, MRI is not feasible, and diagnoses were therefore based exclusively on ultrasound examination. Finally, data collection relied on patient-completed questionnaires rather than national registries; however, patients were assisted by trained healthcare personnel to minimize bias and reduce over- or underestimation of the reported events.

## 5. Conclusions

This two-season study offers one of the first focused epidemiological descriptions of distal upper limb injuries in skiing and snowboarding from a high-volume alpine trauma center. These injuries make up a significant portion of ski-related trauma, with wrist fractures and UCL injuries being the predominant patterns.

Our findings indicate a clear pattern: slow-slope falls predispose to wrist fractures, while high-speed slopes expose skiers to soft tissue injuries, particularly UCL thumb lesions. Notably, female patients demonstrated a higher risk of complex wrist fractures, independent of environmental factors—underscoring an urgent need for targeted prevention strategies in this overlooked area of alpine trauma.

Injury prevention should therefore include specific training on fall techniques, improved ski pole ergonomics, awareness campaigns across all skill levels and sex and closing the gap in the use of protective gear. These strategies, supported by future multicenter research, could lessen the impact of these common but often underestimated injuries.

## Figures and Tables

**Table 1:** Patient characteristics in total sample.

Characteristic	Total (*n* = 195)
Age, mean ± SD (years)	40.4 ± 17.7
BMI, mean ± SD (kg/m^2^)	23.5 ± 3.7
Hours of activity/day, mean ± SD	2.4 ± 1.7
Days of activity/season, mean ± SD	9.1 ± 10.8
**Sex, *n* (%)**	
Male	92 (47.2)
Female	103 (52.8)
**Driving skills, *n* (%)**	
Novice	42 (21.5)
Good	95 (48.7)
Expert	52 (26.7)
Professional athlete	6 (3.1)

Values are presented as mean ± SD or *n* (%). Abbreviations: BMI, body mass index; SD, standard deviation.

**Table 2:** Environmental Context and Equipment Characteristics in total sample.

Characteristic	Total (*n* = 195)
**Slope difficulty, *n* (%)**	
Blue	87 (44.6)
Red	79 (40.5)
Black	19 (9.7)
Off-piste	9 (4.6)
Snowpark	1 (0.5)
**Snow quality, *n* (%)**	
Well prepared	129 (66.2)
Ice	28 (14.4)
Buckle	27 (13.8)
Fresh snow	5 (2.6)
Off track	6 (3.1)
**Weather conditions, *n* (%)**	
Sunny	135 (69.2)
Cloudy	39 (20.0)
Fog/Poor visibility	9 (4.6)
Snow	12 (6.2)
**Equipment ownership, *n* (%)**	
Rented	114 (58.5)
Owned	81 (41.5)
**Type of equipment, *n* (%)** [a]	
Race carver	46 (23.6)
All-round/Easy carver	70 (35.9)
Slalom carver	18 (9.2)
Touring ski	34 (17.4)
Twin tip	4 (2.1)
Other	20 (10.3)
Snowboard	1 (0.5)

Values are presented as *n* (%). [a] Two cases missing equipment type information.

**Table 3:** Accident and Injury Characteristics in total sample.

Characteristic	Total (*n* = 195)
**Cause of accident, *n* (%)**	
Mistake	128 (65.6)
Jump	13 (6.7)
Collision	29 (14.9)
Cause of matter	10 (5.1)
Chairlift	4 (2.1)
Fatigue	11 (5.6)
**Binding release, *n* (%)**	
Opened	62 (31.8)
Not opened	133 (68.2)
**Injury site, *n* (%)**	
Arm	3 (1.5)
Finger	26 (13.3)
Hand	69 (35.4)
Wrist	97 (49.7)
**Injury side, *n* (%)**	
Left	97 (49.7)
Right	98 (50.3)

Values are presented as *n* (%).

**Table 4:** Patient characteristics in Fracture and non-Fracture groups.

Characteristic	Fracture (*n* = 96)	Non-Fracture (*n* = 99)	*p*-Value
Total, *n*	96	99	
Age, mean ± SD	38.7 ± 17.1	42.1 ± 18.2	0.183
BMI, mean ± SD	23.5 ± 3.6	23.5 ± 3.7	0.988
Hours activity, mean ± SD	2.2 ± 1.6	2.6 ± 1.7	0.065
Days activity, mean ± SD	7.9 ± 8.0	10.2 ± 12.8	0.131
Sex, *n* (%)			1.000
– Male	45 (46.9%)	47 (47.5%)	
– Female	51 (53.1%)	52 (52.5%)	
Driving skills, *n* (%)			0.090
– Novice	27 (28.1%)	15 (15.2%)	
– Good	40 (41.7%)	55 (55.6%)	
– Expert	25 (26.0%)	27 (27.3%)	
– Professional athlete	4 (4.2%)	2 (2.0%)	

Values are presented as mean ± SD or *n* (%). Abbreviations: BMI, body mass index; SD, standard deviation.

**Table 5:** Environmental Context and Equipment Characteristics in Fracture and non-Fracture groups.

Characteristic	Fracture (*n* = 96)	Non-Fracture (*n* = 99)	*p*-Value
– Blue			<0.001
– Red	54 (56.2)	33 (33.3)	
– Black	30 (31.2)	49 (49.5)	
– Off piste	11 (11.5)	8 (8.1)	
– Snowpark	0 (0.0)	9 (9.1)	
**Snow quality, *n* (%)**	1 (1.0)	0 (0.0)	
– Well prepared			0.0654
– Ice	64 (66.7)	65 (65.7)	
– Buckle	15 (15.6)	13 (13.1)	
– Fresh snow	16 (16.7)	11 (11.1)	
– Snowpark	1 (1.0)	4 (4.0)	
– Off track	0 (0.0)	0 (0.0)	
**METEO conditions, *n* (%)**	0 (0.0)	6 (6.1)	
– Sunny			0.7103
– Cloudy	66 (68.8)	69 (69.7)	
– Fog/poor visibility	20 (20.8)	19 (19.2)	
– Snow	3 (3.1)	6 (6.1)	
**Equipment, *n* (%)**	7 (7.3)	5 (5.1)	
– Rented			0.7441
– Owned	55 (57.3)	59 (59.6)	
**Type of equipment, *n* (%)**	41 (42.7)	40 (40.4)	
– Race carver	[b]		0.0637
– All-round/Easy-Carver	26 (27.1)	20 (20.2)	
– Slalom Carver	29 (30.2)	41 (41.4)	
– Toure ski	7 (7.3)	11 (11.1)	
– Twin Tip	18 (18.8)	16 (16.2)	
– Other	0 (0.0)	4 (4.0)	
– Snowboard	14 (14.6)	6 (6.1)	
– Blue	0 (0.0)	1 (1.0)	

Values are presented as *n* (%). [b] Two cases missing equipment type information in fracture group.

**Table 6:** Accident and Injury Characteristics in Fracture and Non-Fracture groups.

Characteristic	Fracture (*n* = 96)	Non-Fracture (*n* = 99)	*p*-Value
**Cause, *n* (%)**			0.5692
– Mistake	66 (68.8)	62 (62.6)	
– Jump	6 (6.2)	7 (7.1)	
– Collision	14 (14.6)	15 (15.2)	
– Cause of matter	4 (4.2)	6 (6.1)	
– Chairlift	3 (3.1)	1 (1.0)	
– Fatigue	3 (3.1)	8 (8.1)	
– Other	0 (0.0)	0 (0.0)	
**Binding open, *n* (%)**			0.4377
– Opened	28 (29.2)	34 (34.3)	
– Not Opened	68 (70.8)	65 (65.7)	
**Injury site, *n* (%)**			**<0.001**
– Arm	2 (2.1)	1 (1.0)	
– Finger	8 (8.3)	18 (18.2)	
– Hand	20 (20.8)	49 (49.5)	
– Wrist	66 (68.8)	31 (31.3)	
**Side, *n* (%)**			0.1612
– Left	51 (53.0)	46 (46.5)	
– Right	45 (47.0)	53 (53.5)	

Values are presented as *n* (%).

**Table 7:** Patient characteristics in Good-Fracture and Bad-Fracture groups.

Characteristic	Good (*n* = 47)	Bad (*n* = 16)	*p*-Value
Total, *n*	47	16	
Age, mean ± SD (years)	35.8 ± 17.5	40.1 ± 11.7	0.269
BMI, mean ± SD (kg/m^2^)	23.2 ± 3.4	23.5 ± 3.1	0.783
Hours of activity, mean ± SD	2.3 ± 1.8	2.4 ± 1.9	0.823
Days of activity, mean ± SD	7.7 ± 9.1	6.9 ± 5.1	0.689
Driving duration, mean ± SD	13.0 ± 13.1	14.8 ± 12.2	0.632
Sex, *n* (%)			**<0.005**
– Male	22 (46.8%)	4 (25.0%)	
– Female	25 (53.2%)	12 (75.0%)	
Driving skills, *n* (%)			0.230
– Novice	20 (42.6%)	3 (18.8%)	
– Good	16 (34.0%)	8 (50.0%)	
– Expert	11 (23.4%)	5 (31.2%)	
– Professional athlete	0 (0.0%)	0 (0.0%)	

Values are presented as mean ± SD or *n* (%). Abbreviations: BMI, body mass index; SD, standard deviation.

**Table 8:** Environmental Context and Equipment Characteristics Good-Fracture and Bad-Fracture groups.

Characteristic	Good (*n* = 47)	Bad (*n* = 16)	*p*-Value
Slope difficulty, *n* (%)			0.7521
– Blue	29 (61.7%)	12 (75.0%)	
– Red	14 (29.8%)	3 (18.8%)	
– Black	3 (6.4%)	1 (6.2%)	
– Off slope	0 (0.0%)	0 (0.0%)	
– Snowpark	1 (2.1%)	0 (0.0%)	
Snow quality, *n* (%)			0.6019
– Well prepared	30 (63.8%)	13 (81.2%)	
– Ice	4 (8.5%)	1 (6.2%)	
– Buckle	12 (25.5%)	2 (12.5%)	
– Fresh snow	1 (2.1%)	0 (0.0%)	
– Snowpark	0 (0.0%)	0 (0.0%)	
– Off track	0 (0.0%)	0 (0.0%)	
METEO conditions, *n* (%)			0.9015
– Sunny	32 (68.1%)	11 (68.8%)	
– Cloudy	10 (21.3%)	3 (18.8%)	
– Fog/poor visibility	1 (2.1%)	0 (0.0%)	
– Snow	4 (8.5%)	2 (12.5%)	
Equipment, *n* (%)			0.5141
– Rented	25 (53.2%)	7 (43.8%)	
– Owned	22 (46.8%)	9 (56.2%)	
Type of equipment, *n* (%)			0.5797
– Race carver	13 (27.7%)	4 (25.0%)	
– All-round/Easy-Carver	9 (19.1%)	3 (18.8%)	
– Slalom Carver	4 (8.5%)	1 (6.2%)	
– Toure ski	15 (31.9%)	3 (18.8%)	
– Twin Tip	0 (0.0%)	0 (0.0%)	
– Other	6 (12.8%)	5 (31.2%)	
– Snowboard	0 (0.0%)	0 (0.0%)	

Values are presented as *n* (%).

**Table 9:** Accident and Injury Characteristics in Good Fracture and Bad Fracture groups.

Characteristic	Good (*n* = 47)	Bad (*n* = 16)	*p*-Value
Cause, *n* (%)			0.8518
– Mistake	37 (78.7%)	12 (75.0%)	
– Jump	1 (2.1%)	1 (6.2%)	
– Collision	3 (6.4%)	1 (6.2%)	
– Cause of matter	3 (6.4%)	1 (6.2%)	
– Chairlift	1 (2.1%)	1 (6.2%)	
– Fatigue	2 (4.3%)	0 (0.0%)	
– Other	0 (0.0%)	0 (0.0%)	
Binding open, *n* (%)			0.4795
– Opened	13 (27.7%)	3 (18.8%)	
– Not Opened	34 (72.3%)	13 (81.2%)	
Type of injury, *n* (%)			0.5564
– Fracture	47 (100%)	16 (100.0%)	
Injury site, *n* (%)			0.5564
– Arm	1 (2.1%)	0 (0.0%)	
– Finger	0 (0.0%)	0 (0.0%)	
– Hand	0 (0.0%)	0 (0.0%)	
– Wrist	46 (97.9%)	16 (100.0%)	
Side of injury, *n* (%)			0.6995
– Left	26 (55.3%)	9 (56.2%)	
– Right	19 (40.4%)	7 (43.8%)	

Values are presented as *n* (%).

**Table 10:** Patient characteristics in UCL Injury.

Characteristic	Value	*p*-Value
Total, *n*	33	
Sex, *n* (%)		0.862
– Male	16 (48.5%)	
– Female	17 (51.5%)	
Age, mean ± SD (years)	43.2 ± 16.8	
BMI, mean ± SD (kg/m^2^)	24.1 ± 3.2	
Hours of activity, mean ± SD	2.9 ± 1.7	
Days of activity, mean ± SD	10.1 ± 8.5	
Skills level, *n* (%)		**<0.001**
– Novice	3 (9.1%)	
– Good	20 (60.6%)	
– Expert	10 (30.3%)	
– Professional athlete	0 (0.0%)	

Values are presented as mean ± SD or *n* (%). Abbreviations: BMI, body mass index; SD, standard deviation.

**Table 11:** Environmental Context and Equipment Characteristics in UCL Injury.

Characteristic	Value	*p*-Value
Slope difficulty, *n* (%)		**<0.001**
– Blue	10 (30.3%)	
– Red	18 (54.5%)	
– Black	3 (9.1%)	
– Off piste	2 (6.1%)	
Snow quality, *n* (%)		**<0.001**
– Well prepared	24 (72.7%)	
– Ice	4 (12.1%)	
– Buckle	4 (12.1%)	
– Fresh snow	1 (3.0%)	
METEO conditions, *n* (%)		**<0.001**
– Sunny	21 (63.6%)	
– Cloudy	8 (24.2%)	
– Fog/poor visibility	2 (6.1%)	
– Snow	2 (6.1%)	
Equipment, *n* (%)		0.117
– Rented	21 (63.6%)	
– Owned	12 (36.4%)	
Equipment type, *n* (%)		**<0.001**
– All-round	17 (51.5%)	
– Race carver	6 (18.2%)	
– Slalom Carver	4 (12.1%)	
– Toure ski	4 (12.1%)	
– Twin Tip	1 (3.0%)	
– Other	1 (3.0%)	

Values are presented as *n* (%).

**Table 12:** Accident and Injury Characteristics in UCL Injury.

Characteristic	Value	*p*-Value
Cause, *n* (%)		**<0.001**
– Mistake	24 (72.7%)	
– Jump	5 (15.2%)	
– Collision	1 (3.0%)	
– Chairlift	1 (3.0%)	
– Fatigue	2 (6.1%)	
Opening binding, *n* (%)		0.117
– Opened	12 (36.4%)	
– Not Opened	21 (63.6%)	
Site of injury, *n* (%)		0.872
– Left	14 (42.4%)	
– Right	19 (57.6%)	

Values are presented as *n* (%).

## Data Availability

All data will be available upon written request to the main author.

## Abbreviations

The following abbreviations are used in this manuscript:

AO	Arbeitsgemeinschaft für Osteosynthesefragen
BMI	Body mass index
UCL	Ulnar collateral ligament
ACL	Anterior cruciate ligament
MCL	Medial collateral ligament
MCPJ	Metacarpophalangeal joints
SD	Standard deviation
*n*	number
SABES	Suedtiroler Sanitaetsbetrieb—Azienda Sanitaria dell’ Alto Adige
